# Factor analysis of the Zung self-rating depression scale in a large sample of patients with major depressive disorder in primary care

**DOI:** 10.1186/1471-244X-8-4

**Published:** 2008-01-14

**Authors:** Irene Romera, Helena Delgado-Cohen, Teresa Perez, Luis Caballero, Immaculada Gilaberte

**Affiliations:** 1 Clinical Research Department, Lilly SA, Madrid, Spain; 2 Department of Statistics and Operational Research III, Complutense University, Madrid, Spain; 3 Psychiatry Department, Hospital Puerta de Hierro, Madrid, Spain

## Abstract

**Background:**

The aim of this study was to examine the symptomatic dimensions of depression in a large sample of patients with major depressive disorder (MDD) in the primary care (PC) setting by means of a factor analysis of the Zung self-rating depression scale (ZSDS).

**Methods:**

A factor analysis was performed, based on the polychoric correlations matrix, between ZSDS items using promax oblique rotation in 1049 PC patients with a diagnosis of MDD (DSM-IV).

**Results:**

A clinical interpretable four-factor solution consisting of a *core depressive *factor (I); a *cognitive *factor (II); an *anxiety *factor (III) and a *somatic *factor (IV) was extracted. These factors accounted for 36.9% of the variance on the ZSDS. The 4-factor structure was validated and high coefficients of congruence were obtained (0.98, 0.95, 0.92 and 0.87 for factors I, II, III and IV, respectively). The model seemed to fit the data well with fit indexes within recommended ranges (GFI = 0.9330, AGFI = 0.9112 and RMR = 0.0843).

**Conclusion:**

Our findings suggest that depressive symptoms in patients with MDD in the PC setting cluster into four dimensions: *core depressive, cognitive, anxiety *and *somatic*, by means of a factor analysis of the ZSDS. Further research is needed to identify possible diagnostic, therapeutic or prognostic implications of the different depressive symptomatic profiles.

## Background

Depression can be manifested as a combination of a wide variety of symptoms: loss of interest, depressed mood, psychic anxiety, somatic anxiety, altered appetite, altered sleep, painful symptoms, etc. [1]. In the primary care (PC) setting, approximately two thirds of patients with depression report somatic symptoms solely as the reason for consultation [1]. Indeed, depression is mostly difficult to recognize in such patients being the major reason for underdiagnosis and undertreatment of depression in PC [1-4].

Traditionally, the heterogeneous symptoms of depression have been grouped into different symptomatic dimensions according to their clinical significance but there is no consensus on how this is best done [1,5]. Hence, depressive symptoms have been grouped into psychological and somatic [1]; into affective, cognitive, vegetative, behavioural, physical and impulsive-control [6,7]; or into affective, somatic and cognitive symptoms [8], etc. However, from a clinical perspective, the grouping of depressive symptoms into symptomatic dimensions is purely intuitive and lacks empirical evidence. There is evidence that patients with different depressive symptom profiles are likely to have different prognosis and therefore might require a different therapeutic approach [9]. Furthermore, depressive symptoms have shown to differentially predict survival in patients with coronary artery disease [10]. Therefore, identifying the symptomatic dimensions of depression is relevant because of their diagnostic and therapeutic implications [3,4].

There are few studies that identify or empirically group depressive symptoms into symptomatic dimensions. Some have analysed the factor structure of commonly used diagnostic instruments for depression, such as the Zung self-rating depression scale (ZSDS) or the Hamilton depression rating scale (HAMD-D), to examine the degree to which the emerged factors represent symptoms clusters and to assess whether the obtained factor structures are or not equivalent across subgroups (age, gender, diagnosis, etc.) [9].

In particular, the factor structure of the ZSDS has been studied in different populations, such as healthy subjects over the age of 65 [11], pregnant women [12], patients with heart disease [10], cancer [13,14] or chronic muscle pain [15], students [16,17], workers [18,19] etc., obtaining different factor structures. To date, and to our knowledge there are no studies examining the factor structure of the ZSDS in patients with depression in the PC setting.

Because of the underdiagnosis and undertreatment of depression in PC [2-4,20,21] as well as the possible future implications of different symptomatic profiles in the prognosis of depression, we believe that studying the symptomatic dimensions in this population is of great interest. We hypothesized that depressive symptoms in patients with MDD would be empirically grouped into symptomatic dimensions and that this grouping would be of clinical significance. For this purpose, we examined the factor structure and the composition of the resulted factors in a large sample of patients with MDD in PC by means of a factor analysis of the ZSDS.

## Methods

The factor analysis presented in the current manuscript is a post-hoc analysis of the data reported in a large cross-sectional epidemiological study conducted on 1150 patients diagnosed with MDD, in accordance with the *Diagnostic and Statistical Manual of Mental Disorders, Fourth Edition *(DSM-IV) criteria [22]. The study was reviewed and approved by the ethical review committee of Puerta de Hierro Hospital in Madrid.

Patients were selected from seventy-nine PC sites widely distributed across Spain. Subjects seeking consultation for whatever reason between April and July 2004 were selected using a systematic procedure based on appointment logs. Other inclusion criteria included being at least 18 years of age and not having any condition that would impede understanding of the study or the informed consent. A signed authorization for the collection and use of clinical data in accordance with standing regulations regarding personal data protection was obtained from all subjects prior to enrolment. Screening for depressive symptoms was performed at a cut-off point of ≥ 3 positive responses on the 9-item scale of depression of the Spanish version of the *Goldberg Anxiety and Depression Scale *(GADS) [23]. According to available data [24] this cut-off yields sensitivity of 0.74 and specificity of 0.93. The confirmation of MDD diagnosis as per the DSM-IV criteria was evaluated by means of the *Mini International Neuropsychiatric Interview *(MINI) [25]. All participating physicians attended a one-day training session prior to study commencement to establish uniform criteria as to the use of the assessment instruments and data collection.

The severity of depression was assessed by the patients using the ZSDS and by physicians using the Clinical Global Impression of Severity (CGI-S). The Zung self-rating depression scale [26] is a self-reported 20-item measure of the symptoms of depression. Items responses are ranked from 1 to 4, with higher scores corresponding to more frequent symptoms. Therefore, for each item, patients have to score according to whether the item has occurred 1 = A little of the time/very rarely/rarely; 2 = Once in a while/some of the time/occasionally; 3 = Good part of the time/very often/often; 4 = Most of the time/always/almost always. Ten items are worded positively and the other 10 are worded negatively. Total scores on the ZSDS do not correspond with a clinical diagnosis of depression but rather indicate the level of depressive symptoms that may be of clinical relevance. It has been established as a valid, reliable instrument in several studies in order to measure depressive symptoms [27-29]. The CGI-S scale is a 7-category scale in which the investigator scores the severity of a patient's mental disorder. Thus 1 = "normal, not depressed;" 2 = "on the border of depression;" 3 = mildly depressed;" 4 = "moderately depressed;" 5 = "notably depressed;" 6 = "severely depressed," 7 = "extremely depressed."

### Exploratory Factor Analysis of the ZSDS

An exploratory factor analysis on the ZSDS scores was used to extract the factor solution. The factor analysis was performed, based on the polychoric correlations matrix between ZSDS items by means of the unweighted least squares method, since the normality supposition was not met [30], and using promax oblique rotation [31]. After rotation items with a loading of at least 0.25 were considered to load significantly onto a particular factor.

The sample was divided into two sub-samples of 75% and 25%. The first sample was used to perform an exploratory factor analysis (exploratory phase) and the second was used to validate the results (validation phase) and to perform a confirmatory factor analysis by means of the unweighted least squares method. We used several indexes such as the Goodness of Fit Index (GFI), which should be greater than 0.90 for good-fitting models, GFI Adjusted for Degrees of Freedom (AGFI), with a value of 0.90 as the cut-off value [32] and Root Mean Square Residual (RMR), the larger the RMR value the less is the fit between the model and the data [30].

Finally, subgroups were analysed by gender, age (≤ 65 years vs. > 65 years), place of residence (rural vs. semi rural vs. urban) and severity of depression groups, using the Clinical Global Impression Severity Scale (CGI-S). CGI values were grouped as follows: mild-moderate: "mildly depressed" (3), "moderately depressed" (4) and "notably depressed" (5); Severe: "severely depressed" (6) and "extremely depressed" (7). To compare these groups, mean estimates were made of each factor, by adding the mean scores of all patients in each factor extracted from the factor model in each group. A variance analysis was performed to detect any differences.

And finally, a descriptive examination of the 20-item ZSDS was performed at a quantitative level, calculating sample means and standard deviations, and also at a categorical level, providing percentages for each response.

## Results

### Descriptive characteristics

Table 1 shows the demographic and clinical characteristics of the patients. It should be noted that the sample was predominantly female (75.2%), 54% of patients were not currently diagnosed with depression by their doctor, and remarkably only 31% of patients were receiving antidepressant treatment.

**Table 1:** Demographic and clinical characteristics of patients (n = 1150)

**Demographic and clinical characteristics**	**Patients with MDD**		
	
	N	n	%
Gender			
Women	1138	856	75.2
Men	1138	282	24.8
Place of residence			
Urban	1142	537	47.0
Semi-urban	1142	384	33.6
Rural	1142	221	19.4
Current diagnosis of depression for this episode	1097	504	45.9

Severity of depression (ZSDS)			
None	1049	42	4.0
Mild	1049	185	17.6
Moderate	1049	355	33.8
Severe/extreme	1049	467	44.5

Current medication			
Analgesics/anti-inflammatories	1150	616	53.6
Benzodiazepines	1149	518	45.1
Antidepressants	1150	356	31.0
Antipsychotics	1150	23	2.0

	**N**	**Mean**	**SD**
	
Age (in years)	1112	55.0	15.4
CGI-S	1121	4.1	1.2

### Prevalence of depressive symptoms according to the ZSDS

**Figure 1:**
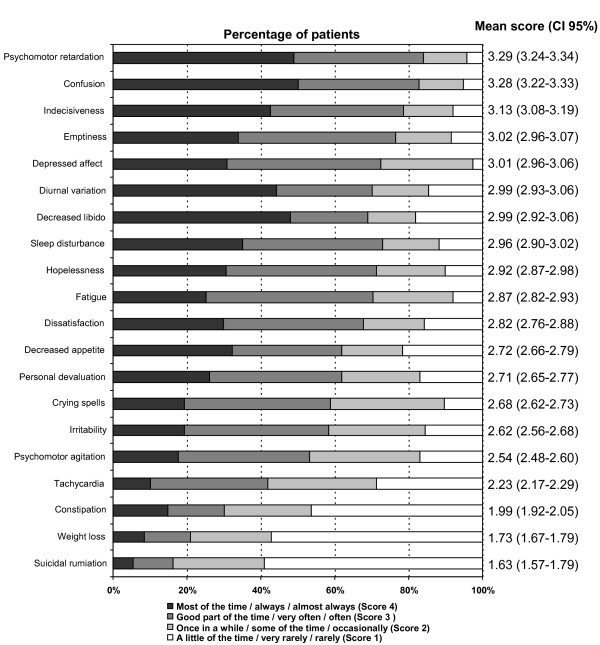
**Characteristics of depressive symptoms according to the Zung Self-Rating Depression Scale (n = 1049)**. The highest score in a single item of the ZSDS is 4 (i.e. most of the time/always/almost always) and the lowest score is 1 (i.e. a little of the time/very rarely/rarely)

A total of 1049 (91.2%) patients completed the questionnaire. The characteristics of depressive symptoms according to the ZSDS are detailed in Figure 1. The highest mean scores of the ZSDS were observed in the following items: psychomotor retardation (mean = 3.29; CI 95%: 3.24–3.34), confusion (mean = 3.28; CI 95%: 3.22–3.33), indecisiveness (mean = 3.13; CI 95%: 3.08–3.19), emptiness (mean = 3.02; CI 95% 2.96–3.07) and depressed affect (mean = 3.01; CI 95% 2.96–3.06). Of note, the most frequent symptoms (psychomotor retardation and confusion) were reported by 50% of patients as present *most of the time/always/almost always *(Figure 1).

The lowest mean scores of the ZSDS were observed in items: suicidal rumiation (mean = 1.63, CI 95%: 1.57–1.68), weight loss (mean = 1.73, CI 95%: 1.67–1.79) and constipation (mean = 1.99, CI 95%: 1.92–2.05). Of these, suicidal rumiation and constipation, the least frequent symptoms were mostly reported as sporadic (a little of the time/very rarely/rarely) (Figure 1).

### Exploratory Factor Analysis of the ZSDS

In the exploratory phase (n = 787), factor extraction resulted in four factors with a significant eigenvalue of at least 1.0 which explained 36.9% of total variance on the ZSDS item intercorrelations. Each factor was composed of 3 to 8 items, with no items loading in more than one factor. After rotation of the four-factor solution, items were considered to load on a factor if the rotated factor loading was at least 0.25. It was then assessed by the research clinician whether the factor solution obtained could have a clinical interpretation (Table 2).

**Table 2:** Pattern of factors after rotation. Values for items with greater weight and greater intercorrelation are highlighted in bold

**Items**	**Factor I**	**Factor II**	**Factor III**	**Factor IV**
18: Emptiness	**0.81020**	0.02694	-0.07882	-0.05805
14: Hopelessness	**0.71806**	-0.01248	0.00358	0.01279
17: Personal devaluation	**0.66729**	0.06068	-0.05545	-0.16968
19: Suicidal rumiation	**0.59081**	-0.05977	0.14953	0.05850
20: Dissatisfaction	**0.56170**	0.21835	-0.12945	0.11518
1: Depressed affect	**0.48840**	0.03523	0.27387	0.06180
6: Decreased libido	**0.39334**	0.11583	-0.11821	0.24162
12: Psychomotor retardation	-0.02329	**0.76309**	-0.01425	0.04242
11: Confusion	-0.00711	**0.62654**	0.10019	-0.00484
16: Indecisiveness	0.27074	**0.35343**	0.03434	-0.01957
10: Fatigue	0.12667	**0.27508**	0.18488	0.06714
13: Psychomotor agitation	-0.06231	0.07960	**0.53666**	0.01544
15: Irritability	0.01450	0.10047	**0.52265**	0.08437
3: Crying spells	**0.38120**	0.00120	0.33934	0.08437
4: Sleep disturbances	0.03951	0.01288	**0.31263**	0.20566
5: Decreased appetite	-0.00035	0.12560	-0.13281	**0.55308**
7: Weight loss	-0.04474	-0.02156	0.05212	**0.53798**
9: Tachycardia	0.13362	-0.07156	0.25808	**0.26486**

**Figure 2:**
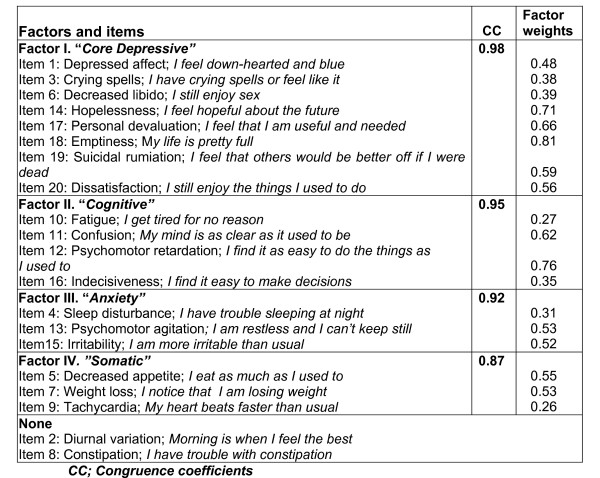
Factor solution of the Zung Self-Rating Depression Scale.

The first factor (factor I) accounting for 23.8% of the scale variance, was composed of 8 items: depressed affect (item 1), crying spells (item 3), decreased libido (item 6), hopelessness (item 14), personal devaluation (item 17), emptiness (item 18), suicidal rumiation (item 19) and dissatisfaction (item 20). The second factor (factor II) was composed of 4 items: confusion (item 11), psychomotor retardation (item 12), indecisiveness (item 16) and fatigue (item 10). This factor accounted for 5.8% of variance. The third factor (factor III), which accounted for 3.7% of variance, consisted of 3 items: sleep disturbances (item 4), psychomotor agitation (item 13) and irritability (item 15). Finally, factor IV, accounting for 3.5% of variance, consisted of 3 items: decreased appetite (item 5), weight loss (item 7) and tachycardia (item 9) (Figure 2).

According to the polychoric correlations matrix, all items except diurnal variation (item 2) and constipation (item 8), presented high intercorrelations with items within the same factor (Table 2). The items relating to diurnal variation and constipation were eliminated from the factor analysis because of their low correlation with the other items.

The four-factor solution was validated and an identical factor solution was obtained with high congruence coefficients (CC) for each factor: 0.98 (factor I), 0.95 (factor II), 0.92 (factor III) and 0.87 (factor IV) (Figure 2). After this, a second model was run to obtain a 3-factor solution. The resulted 3-factor solution was less optimal since it accounted for less of the total variance (33.3%), presented items loading in more than one factor and items with very low factor weights (< 0.25), and also it was much less clinically meaningful than the 4-factor structure.

### Confirmatory Factor Analysis

The obtained indexes for the 4-factor model were GFI = 0.9330, AGFI = 0.9112 and RMR = 0.0843, and for the 3-factor model were GFI = 0.9164, AGFI = 0.8917 and RMR = 0.0942. Examination of the fit indexes indicated that both models came close to fitting the data however the 4-factor model had better goodness-of-fit coefficients. Besides the 3-factor model did not achieve an acceptable AGFI value whereas the 4-factor model did.

Given the large sample size, the stability of the final four factor solution was determined by repeating exploratory factor analysis of ZSDS items separately on females and males and on patients aged ≤ 65 years and > 65 years, respectively. This analysis showed an identical factor solution in females (mean CC: 0.98); in males (mean CC: 0.94) and in patients aged ≤ 65 years (mean CC: 0.99). However, for patients >65 years, very low CCs were obtained for factor IV.

### Comparison of factor scores by demographic and clinical characteristics

A post-hoc analysis revealed that females had significantly higher scores in factor III (sleep disturbances, psychomotor agitation and irritability) than males (p < 0.001). The same comparison between age groups showed that patients over the age of 65 presented significantly higher scores in factor I (depressed affect, crying spells, decreased libido, hopelessness, personal devaluation, emptiness, suicidal rumination and dissatisfaction) and IV (decreased appetite, weight loss and tachycardia) than those under 65 years (p < 0.001 and p = 0.0002, respectively). Finally, when comparing factor I scores in patients from rural vs. urban settings significantly higher factor scores were observed in patients from a rural or semi-rural setting (p = 0.0005). There were no other significant differences between factor scores and gender, age, place of residence and severity of depression.

## Discussion

The four factors extracted from the factor analysis were interpreted, according to the nature of the symptoms in each factor, as:*core depressive factor *(Factor I), *cognitive factor *(Factor II), *anxiety factor *(Factor III) and *somatic factor (Factor IV)*.

The *core depressive factor *appears to primarily reflect emotional or affective symptoms of depression, such as depressed affect, suicidal rumiation, dissatisfaction and personal devaluation. Other studies have identified a similar factor but used different labels, "*manifest depressed mood" *[13], *"general depression" *or *"negative affect" *being depressed mood the core symptom in all of them [33]. Factor I has the greatest weight, accounting for 23.8% of the ZSDS variance, which suggests that factor I symptoms are more specific of depression and therefore more relevant when diagnosing depression in PC patients. This factor includes two symptoms that are essential in order to diagnose depression such as depressed mood and loss of interest or ability to enjoy things as reflected in items 1: "I feel down-hearted and blue," 14: "I feel hopeful about the future" and 20: "I still enjoy the things I used to do." It should be noted that the "decreased libido" item is included in factor I. In other studies this item was also associated with core depressive symptoms [6,11] possibly due to the way in which this item is worded "I still enjoy sex" that would reflect the loss of sexual drive.

Factor II appears to reflect symptoms related to difficulty in concentrating/decision-making, confusion or loss of mental clarity, and psychomotor retardation. These items seem to reflect altered cognitive function possibly due to decreased concentration and speed of response. Indeed, this is why this factor has been interpreted as *cognitive*. Sugawara M and colleagues found a similar grouping of ZSDS items (confusion, psychomotor retardation and indecisiveness) in a sample of women during pregnancy and the post-partum period [12].

Interestingly, unlike other factor studies on the ZSDS [11-16,18,19] we have found a factor (III) that groups items associated with typical symptoms of anxiety disorders (irritability, psychomotor agitation, restlessness, and sleep disturbances) in particular generalised anxiety disorder [33]. For this reason factor III has been labelled as *anxiety factor*. Despite methodological differences, these symptoms (irritability, psychomotor agitation and sleep disturbances) were also grouped together in another study conducted on a combined sample of PC attenders and community residents [5]. It is possible that, unlike other factor analyses of the ZSDS, we have identified an anxiety dimension due to the fact that our sample comes from a PC setting, with a predominance of females (75.2%). This is consistent with previous reports showing that *anxious depression *is more likely to be present amongst women and in PC settings [34]. With regard to factor IV, interpreted as a *somatic factor *(decreased appetite, weight loss and tachycardia), our findings are consistent with those reported by other studies [10,13,17].

Collectivelly, these results indicate that the ZSDS provides a factor structure of clinical relevance in this patient population. However, since the factor structure of this scale has not been examined in PC patients with depression before further studies are needed in order to confirm these results. When interpreting this factor solution it is worth noting that our sample is a PC population with a diagnosis of current MDD episode present only in less than half of patients (45.9%) and only 31% of depressed patients were taking antidepressant medication. This reflects the underdiagnosis and undertreatment of MDD in PC already addressed at length by other studies [35-38].

Previous factor analysis studies on ZSDS have proposed four-, three- or two- factor models with differences in the type and number of items loaded in each factor. This may be due to the fact that those studies were conducted on different sample populations. It is not surprising to find diverse clusters of symptoms as the profile of depressive symptoms also differs across miscellaneous populations [9,39]. As previously mentioned similar studies need to be conducted on patients with MDD in a PC setting in order to confirm our results and to clarify whether or not there is a characteristic profile of symptoms in patients with depression in PC. It would be also interesting to assess the possible diagnostic, therapeutic or prognostic implications of the different symptomatic profiles.

The notably high factor III scores in females indicate that females with MDD in a PC setting are more likely to present depression-related anxiety symptoms than males. However no such differences emerged between gender and factors I, II and IV. In this respect our data confirm previous results reporting a higher prevalence of anxiety symptoms in women than men [40,41]. When comparing factor scores by age groups, we observed that patients over the age of 65 with MDD in a PC setting tend to present more core depressive and somatic symptoms than patients aged 65 and under. These findings support data obtained by other authors suggesting that elderly patients with MDD are more commonly characterised by the manifestation of somatic symptoms than younger patients [42-44]. Finally, we did not find differences with regard to gender or place of residence as expected [1,45], possibly because few somatic items loaded in this factor.

### Limitations

Although this study used a large sample of patients, the results must be interpreted with caution. Considering that the factor solution accounted for only 36.9% of variance in the ZSDS, and that it was decided to exclude items 2 and 8 from the factor analysis because of their low correlation, it would not be appropriate to extract 4 sub-scales from these factors. Extracting four sub-scales would mean that some of the original items, specifically items 9 (tachycardia) and 10 (fatigue) would not be properly accounted for. One limitation in our study is that fatigue loads in the *cognitive *factor but with a low factor weight and an ambiguous clinical interpretation therefore this result should be interpreted with caution.

The obtained factor solutions in other studies accounted for variances ranging from 32–33% [18,19] to 46%–48% [10,13]. Similarly, the low percentages of variability explained by the *anxiety *(III) and *somatic *(IV) factors, 3.7% and 3.5% respectively, may reflect limited stability of those factors. However the stability of the final four factor solution was confirmed since an identical factor structure was extracted in females, males and patients aged ≤ 65. Also there are studies reporting a somatic factor with the same items loading to that factor as we have found.

The cross-sectional character of the study does not allow the identification of the stability of the resulted factors. Further, the lack of a control population without depression makes impossible to draw comparisons with a non-clinical population. Finally, when comparing factor scores according to the demographic and clinical characteristics of patients, we noted significant differences in sample size impairing the statistical power of these analyses.

## Conclusion

To our knowledge, this is the first study that identifies four symptomatic factors of depression in a large sample of patients with MDD in PC. Depressive symptoms in PC patients cluster into *core depressive, cognitive, anxiety *and *somatic *dimensions by means of a factor analysis of ZSDS. However, since the factor structure of this scale has not been previously examined in such population, further investigation is needed in order to confirm these results. Future studies could help to clarify whether there is a characteristic profile of symptoms in patients with depression in PC as well as any possible diagnostic, therapeutic or prognostic implications of the different symptomatic profiles.

## Competing interests

Irene Romera, Helena Delgado-Cohen and Inmaculada Gilaberte are full-time employees of Lilly S.A., Avda. de la Industria, 30. 28108 Alcobendas – Spain.

## Authors' contributions

Author IR, LC and IG. designed the study and wrote the protocol. Authors IR. and HD-C managed the literature searches and analyses. Authors TP undertook the statistical analysis, and authors HD-C, IR and TP. wrote the first draft of the manuscript. All authors contributed to and have approved the final manuscript.

## Pre-publication history

The pre-publication history for this paper can be accessed here:


